# Ultra-purification of Lipopolysaccharides reveals species-specific signalling bias of TLR4: importance in macrophage function

**DOI:** 10.1038/s41598-020-79145-w

**Published:** 2021-01-14

**Authors:** Matthew Stephens, Shan Liao, Pierre-Yves von der Weid

**Affiliations:** 1 Department of Physiology and Pharmacology, Inflammation Research Network, Snyder Institute for Chronic Diseases, Cumming School of Medicine, University of Calgary, Alberta, Canada; 2 Department of Microbiology, Immunology & Infectious Disease, Inflammation Research Network, Snyder Institute for Chronic Diseases, Cumming School of Medicine, University of Calgary, Alberta, Canada

**Keywords:** Innate immune cells, Monocytes and macrophages, NF-kappaB, Pattern recognition receptors

## Abstract

TLR4 location, and bacterial species-derived lipopolysaccharides, play a significant role in the downstream activation of transcription factors, accessory molecules, and products. Here, this is demonstrated through the use of classically-activated and alternatively-activated macrophages. We show that, when polarized, human macrophages differentially express and localize TLR4, resulting in biased recognition and subsequent signalling of LPS derived from *Pseudomonas aeruginosa, Escherichia coli, and Salmonella enterica*. Analysis of activation demonstrated that in classically activated macrophages, *P. aeruginosa* signals from the plasma membrane via TLR4 to p65 dependent on TAK1 and TBK1 signalling. *E. coli* signals dependent or independent of the endosome, utilizing both TAK1- and TBK1-signalling to induce P65 and IRF3 inducible genes and cytokines. *S. enterica* however, only induces P65 and IRF3 phosphorylation through signalling via the endosome. This finding outlines clear signalling mechanisms by which innate immune cells, such as macrophages, can distinguish between bacterial species and initiate specialized responses through TLR4.

## Introduction

Chronic inflammatory diseases, such as inflammatory bowel disease (IBD), is driven in part by a dysregulated immune response to the host microbiota. This dysbiosis, can be driven by infection or through a bloom of naturally commensal, turned pathogenic bacteria, known as pathobionts. Common commensal bacteria found within the human population that can undergo this switch include, but are not limited to, *Pseudomonas aeruginosa*, *Escherichia coli*, and *Salmonella enterica*^1^. Through adaptive signalling mechanisms and increased virulence factors these bacteria can become problematic with; increased attachment, biofilm formation, and invasiveness^2^. Therefore, being able to distinguish between these non-threatening commensal microbes and those that would cause harm to the host, seems to be an obvious characteristic the body should have developed. However, to current literature understanding, little is known about how the host innate immune system achieves this disparity. A key component of the innate immune cells’ arsenal, involved in the sensing of the plethora of microbial components, is the expression of a wide array of pattern recognition receptors (PRRs).

Within the superfamily of PRRs, a large and well-characterised family are Toll-like receptors (TLRs), which are found distributed widely on the plasma membrane surface, or bound within organelles of many innate immune cells. Current evidence demonstrates that all TLRs, excluding the endosomal dsRNA receptor TLR3, signal through a myeloid differentiation primary response 88 (MyD88)-dependent pathway.

The expression and localization of the LPS receptor, TLR4, is tightly regulated in order to limit the gross-induction of pro-inflammatory mediators through commensal microbial presence, a tolerogenic process^3^. Canonically, the vast majority of TLR4 is expressed on the cell surface membrane where it can sense extracellular bacterial LPS, inducing a cascade event, culminating in the induction of nuclear factor kappa-light-chain-enhancer of activated B cells (NF-κB)-driven cytokines. However, on occasion TLR4 after endocytosis of the receptor/ligand complex from the cell surface, can utilise TIR-domain-containing adapter-inducing interferon-β (TRIF) resulting in an interferon regulatory factor 3 (IRF3) induced type-I interferon response^4^.

Many commensal bacteria species that colonise the intestine are susceptible to being overwhelmed by opportunistic infectious bacteria or, pressured into display a more pathogenic phenotype through environmental stresses^5^. All known gram-negative bacteria express high levels of species-specific LPS within their cell wall outer membrane structure, whose length can be controlled through a process known as phase-shift regulation. The purpose of this phenomenon is debated; however, we and many others believe that in addition to an energy conservation process, LPS shortening and lengthening can aid in bacterial adherence, pathogenicity, and visibility to the immune system^6^.

The aforementioned; *E. coli, S. enterica and P. aeruginosa* are three commensal bacterial strains with serotypes (variants) that have pathogenic characteristics. Due to their LPS content, they all have the ability to activate TLR4, although their infection characteristics (such as where and how they infect) raise questions as to what cells would, and could, recognise them. At the top of this list are macrophages. Both peripherally derived and resident macrophages have the potential to recognise LPS through their abundant expression of TLR4. It should also be noted that in inflammatory situations, such as those found in chronic inflammatory diseases and bacterial infection, their phenotype changes. This phenotypic shift could theoretically further modulate TLR4 dynamics. Whilst there is mounting evidence suggesting altered activation of TLR4 by different species of bacteria, the exact causal agent from the system within live bacteria, is still only theorized and debated.

Macrophages are key players in the innate immune system and during host-defence to invasive pathogens. They respond via recognition, phagocytosis, and participation to the immuno-inflammatory response. Macrophages are plastic cells that can be subdivided into a number of categories based on: morphology, function, and observed purpose. The two most commonly classically described, and widely accepted macrophages sub-types are the pro-inflammatory classically activated M1 (early inflammatory phase) and alternatively-activated resolving/reparative M2 (late inflammatory phase). These terminal differentiation pathways, originally named to mimic the TH1/TH2 paradigm studied around that time^7–9^, can be skewed in phorbol 12-myristate 13-acetate (PMA)-treated monocytes, through co-stimulation with a variety of factors. Commonly, incubation of cultured macrophages with LPS derived from *E. coli* either alone, or alongside IFNγ, will push toward a classically activated M1 phenotype, while co-stimulation with IL-4 and IL-13 are known to influence macrophage polarization to the M2, alternative state. This phenomenon has been shown in more recent years to be reversible and switchable, further demonstrating the plastic nature of the macrophage^10,11^. It is more widely acknowledged now that macrophage cover a spectrum of phenotypes although the overarching M1 and M2 standards are still widely referred to (reviewed in^12^. Macrophages express high levels of PRRs, including TLR4 and respond to LPS producing a wide array of cytokines and chemokines. Interestingly, macrophage polarity in chronic disease progression and resolution is of great interest, as many experimental therapies include shifting these phenotypes from inflammatory to reparative.

Growing evidence suggest that polysaccharides from natural sources have potential as immunomodulators with pharmacological application, but a complete report of bacterially derived LPS is poorly described and widely neglected and many may have beneficial effects^13^. The overall goal of this study was to elucidate species specific LPS recognition and subsequent induction of inflammatory pathways within polarized classically- and alternately-activated macrophages.

## Materials and methods

### Reagents

All reagents were purchased for their cell-culture approved abilities. Products that did not have quantification of endotoxin levels were exposed to HEK293-TLR4 reporter cell lines in the concentrations and times used for all assays to screen for contamination. All products were additionally tested and found to be endotoxin low (EU < 0.01) or undetectable using the Pierce Chromogenic LAL endotoxin quantification assay (Cat No: 88282) as per manufacturer’s instructions. Companies, catalogue numbers, and concentrations used are documented as required throughout the text. Purified LPS of *Salmonella enterica* (L5886-10MG), *Pseudomonas aeruginosa* (L9143-10MG) and *Escherichia coli* O127:B8 (L3129-10MG) were purchased from Sigma Aldrich, reconstituted in endotoxin free cell culture water and made uniformly homogenous through vigorous vortex-mixing for 10 min. Stocks were stored in glass scintillation vials at − 80 °C until use. Chloroquine diphosphate (Cat No: C6628) and TAKINIB (Cat No: SML2216) were purchased from Sigma Aldrich and reconstituted as per manufacturer’s guidelines. MRT67307 (Cat ref: inh-mrt) was purchased from Invivogen and reconstituted as per manufacturer’s instruction and stored at − 80 °C until use.

### Ultra-purification of LPS

Following purification methods described by Hirschfield et al.^14^, ultrapure LPS was obtained for use in all further experiments. In brief, purchased LPS was first resuspended in endotoxin-free water supplemented with 0.2% triethylamine (TEA). Deoxycholate was added to the solution (final concentration of 0.5%) followed immediately by 500 µl of water-saturated phenol. Samples were vortexed for 5 min, incubated on ice for 5 min, then centrifuged at 4 °C for 2 min at 10,000×*g*. The aqueous (top) layer was transferred into a fresh tube and the phenol (lower) phases were pooled and re-extracted with 1 ml of water-saturated phenol. The aqueous phases were adjusted to 75% ethanol and 30 mM sodium acetate and precipitated for at least 1 h at − 20 °C. Precipitates were resuspended in the original volume of 0.2% TEA with 100% recovery being assumed. Samples were diluted for further use in cell grade water before cell-stimulation.

### Cell culture

Human THP-1 monocytic cell lines were maintained in RPMI supplemented with 10% FCS and 1% Pen/Strep according to standard culture protocols. Cells were split every 4 days to maintain proliferative capacity and prevent overgrowth of cells. All cells were maintained in a 37 °C incubator, 5% CO_2_ atmosphere. Cells were differentiated into resting (M0) Macrophages using 50 ng/ml PMA for 48 h before being pushed to a classically activated (M1) or alternate (M2) phenotype. M1 phenotype was achieved through stimulation of M0 resting macrophages with 50 ng/ml recombinant IFNγ (Peprotech cat # 300-02) for 24 h before stimulation. M2 macrophages were differentiated by stimulating resting M0 macrophages with 25 ng/ml recombinant human IL-4 and IL-13 (Peprotech cat # 200-04 and # 200-13) for 24 h.

### Flow cytometry

Isolated macrophages were blocked for non-specific antibody binding in FACS wash buffer (PBS pH 7.2, 5% Fetal calf serum, and 0.1% Sodium azide) containing Fc block for 5 min on ice. Cells were then incubated with cell surface staining antibodies CD14 Cat #367109 Clone 63D3, Biolegend, CA, USA) or TLR4 (Cat #312802 Clone HTA125, Biolegend, CA, USA) or equivalent concentrations of isotype and fluorophore matched controls (Cat# 400111 and 400257, Biolegend, CA, USA) on ice for 30 min, kept dark at 4 °C. Cells were then washed in 1 ml of FACS wash buffer, centrifuged at 800×*g* for 3 min at 4 °C on a benchtop centrifuge, and fixed in FACS wash buffer containing 0.5% (v/v) formalin. Cells were then passed through 100 µm filter topped FACS tubes before analysis using the BD FACS Canto (BD, USA). To study the intracellular/total protein expression, cells were permeabilised after the blocking stage, using permeabilising FACS wash buffer (FACS wash buffer supplemented with 1% w/v Saponin (Sigma, USA)) for 30 min, on ice, in the dark; anti-TLR4 antibody or isotype controls were added and incubated on ice for 30 min in the dark. Cells were then processed as previously detailed.

### Immunofluorescence

1 × 10^5^ THP-1 monocytes were differentiated into resting M0 macrophages in 8 well chamber slides before being pushed to an M1 or M2 phenotype as described above. Cells were then fixed in 4% Paraformaldehyde for 30 min and permeabilised in 2% bovine serum albumin in PBST (Triton X100, 0.3%) for 1 h before staining. Cells were stained with purified anti-human CD284 (TLR4) antibody (Cat #312802 Clone HTA125, Biolegend, CA, USA) at 100 µg/ml overnight at 4 °C, washed 3 times in PBST for 10 min on a rocker and then stained with Rat anti-mouse secondary antibody (100 µg/ml) for 2 h at room temperature on an orbital plate rocker, kept in the dark. Cells were counterstained and mounted in DAPI containing Vectorshield (Cat #H-1200, Vector Laboratories, USA) and installed with a coverslip for imaging. Images were acquired using a Leica SP8 inverted confocal microscope courtesy of the Diane & Irving Kipnes Lymphedema imaging Suite.

### qPCR

The total RNA isolated from given samples was purified using the QIAGEN RNA total cleanup kits as per manufacturers instruction. One hundred nanogram of the RNA was converted using EvaGreen RT conversion kit in a gradient thermocycler as per manufacturers description (ABMbio, USA). One nanogram of the converted cDNA samples was added to Bright-Green SYBR qPCR master-mix and qPCR analysis was performed in an ABI StepOne Plus PCR system. The PCR conditions were as follows: 95 °C for 1 min followed by 40 cycles at 95 °C for 30 s, 60 °C for 30 s and 72 °C for 30 s, and with a final round of extension at 72 °C for 10 min^15^. Sequences of primers used are detailed in Table 1.

**Table 1:** qPCR genes of interest, sense and antisense primer sequence and amplicon size.

Gene	Forward primer 5′-3’	Reverse primer 5′-3’	Size	Refs.
CXCL10	GAAAGCAGTTAGCAAGGAAAGGTC	ATGTAGGGAAGTGATGGGAGAGG	120	^16^
CCR7	TGGTGGTGGCTCTCCTTGTC	TGTGGTGTTGTCTCCGATGTAATC	83	^16^
IL-12	AAAGGACATCTGCGAGGAAAGTTC	CGAGGTGAGGTGCGTTTATGC	128	^16^
CD206	ACCTCACAAGTATCCACACCATC	CTTTCATCACCACACAATCCTC	213	^16^
CD163	GTCGCTCATCCCGTCAGTCATC	GCCGCTGTCTCTGTCTTCGC	114	^16^
CCL17	GAGCCATTCCCCTTAGAAAG	AGGCTTCAAGACCTCTCAAG	172	^16^
NFκB (RELA)	CCAGACCAACAACAACCCCT	TCACTCGGCAGATCTTGAGC	197	^17,18^
IRF3	TCTGCCCTCAACCGCAAAGAAG	TACTGCCTCCACCATTGGTGTC	151	^17^

### ELISA

Enzyme linked immunosorbent assays (ELISA) were performed according to the manufacturer’s instructions. In short 100 µl of primary antibody was incubated on ELISA high-binding 96-well plates for 2 h at RT under agitation at the recommended dilution. Unbound antibody was removed and washed three times with PBST (0.1% Tween-20) for 5 min, each wash performed on an orbital shaker. Non-specific binding was then blocked using 200 µl 2% BSA in PBST for 1 h at RT on the same orbital shaker. Next, standards along with conditioned media from stimulated cells, diluted as necessary, were added to precoated and blocked ELISA plates for 24 h at 4 °C. Unbound product was washed off in the same manner as before. Diluted secondary HRP-conjugated antibodies were then added to the plate and incubated for 1 h and unbound fractions were again removed and the plate washed. 100 µl of TMB substrate was then added to each well and samples were left to develop until the third standard was visible, after which developing was halted by adding stop solution (0.1 N HCl). All plates were read using a Spectramax plate reader at 405 nm and concentrations of cytokines determined in relation to the standard curve.

### Immunoblotting

THP-1 monocytes (1 × 10^6^ cells/well) were differentiated into M1 macrophages as previously described in 6 well tissue culture plates. Samples were stimulated as mentioned and cells were lysed immediately in NP-40 lysis buffer (containing protease inhibitor cocktail, sodium fluoride, sodium orthovanadate, Triton X-100). Samples were then sonicated to ensure complete lysis and dissociation of proteins and clarified at 12,000×*g* for 10 min at 4 °C. Protein concentration was then confirmed and normalised using the Pierce BCA assay and 10 µg protein was loaded into each well. Samples were separated on a 4–20% SDS–polyacrylamide gradient gel (Biorad, USA) at 120 V for 90 min or until the ladder was resolved as required. Samples were then transferred to a nitrocellulose membrane; transfer efficiency was confirmed with Ponceau red staining. All membranes were blocked in 5% BSA TBST (0.1% Tween-20) at RT for 2 h. After washing with TBST, the membrane was probed with primary antibodies according to the manufacturer’s instruction (1:1000 in 3% BSA TBST) O/N at 4 °C, washed three times with TBST and further probed with HRP-conjugated secondary antibody (1:5000 in TBST) for 1 hr at RT. The membrane was finally washed three times with TBST and developed using freshly prepared luminol-based detection solution and imaged immediately.

### Graphical design

Graphical depictions were created using BioRender.com.

### Statistical methods

Data are expressed as the mean ± one standard error of the mean (SEM). Experiments were performed as a minimum experimental triplicate. Statistical significance was assessed through the use of a two-tailed unpaired Student’s t-test for parametric data, while the Mann–Whitney test was performed for non-parametric data. Multiple analyses were performed using a one-way Anova with *post-hoc* Tukeys test where indicated. *P < 0.05, **P < 0.01, ***P < 0.001, ****P < 0.0001^15^.

## Results

### Differentiated classically-activated M1 macrophages display internalisation of TLR4

**Figure 1:**
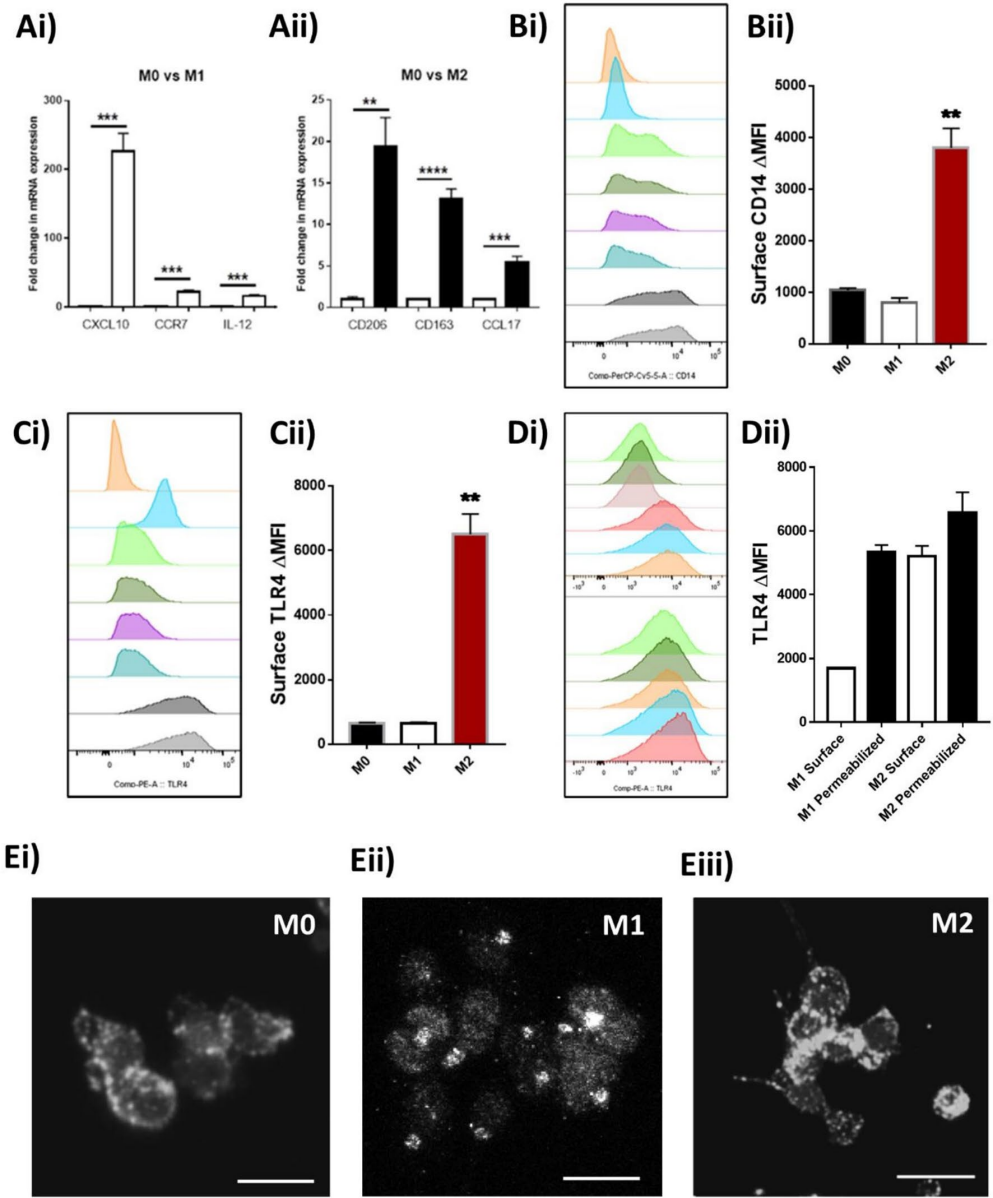
Differentiated M1 and M2 macrophage expression of TLR4 in vitro. PMA differentiated THP-1 monocytes (Resting M0-Macrophages) were stimulated for 24 h with either (50 ng/ml) recombinant human IFNγ (M1 polarization) or 25 ng/ml each of recombinant human IL-4 and IL-13 (M2). Polarity of matching sets of macrophages were initially confirmed by (**A**) qPCR analysis for (**Ai**) M1 markers: CXCL10, CCR7, IL-12 or (**Aii**) M2 markers: CD206, CD163, CCL17. Cell surface expression of (**B**) CD14 or (**C**) TLR4 was determined through flow cytometric analysis. Further analysis of total expression of TLR4 through permeabilization of cells and comparison to cell surface staining reveals (**D**) Abundance of TLR4 on the surface or total (including internal) of M1 and M2 polarized THP-1 macrophages. (**E**) Single colour immunofluorescent confocal microscopy images of TLR4 expression within (**Ei**) M0, (**Eii**) M1, (**Eiii**) M2 macrophages show differential patterns of staining within the cells. Images are a representative of 3 independent experiments. qPCR data is expressed as the mean ± SEM of 3 independent differentiations of THP-1 derived macrophages of passages 5–8. (Histograms: Pink-isotype control, Blue—THP-1 monocyte controls, Green and Dark Green—M0 macrophages, Purple and Blue—M1 macrophages, Dark and light grey—M2 macrophages). Scale bar = 20 µm. Statistical analysis performed on qPCR data was a two tailed multiple unpaired students t-test comparing stimulated to control, **P < 0.01, ***P < 0.001, ****P < 0.0001.

Myeloid lineage cells, such as macrophages are known to express high levels of TLR4 as these innate immune cells can quickly respond to infection through the production of large amounts of chemotactic and inflammatory mediators. We first began by characterising the location and expression of TLR4 in M1 and M2 differentiated THP-1 macrophages through a combination of qPCR, flow-cytometry, and immunofluorescent confocal image analysis. Firstly, analysis of several well-characterised M1 and M2 markers (CXCL10, CCR7, IL-12, CD206, CD163 and CCL17) validated the resting phenotype (Fig. 1A)^16,19^. Flow cytometry revealed that the predominant expression of TLR4 within M1 macrophages was intracellular (68.08 ± 3.17%; Fig. 1D), whilst in M2, expression was primarily on the cell surface (75.99 ± 8.75%; Fig. 1D). This was determined through comparison of surface staining with total staining, via saponin permeabilization. This differential localisation was furthermore confirmed through immunofluorescent imaging of the same macrophages clearly showing a punctate distribution of TLR4 in M1 macrophages (Fig. 1Eii), suggesting compartmentalisation, perhaps within the early-endosome, whilst M0 and M2 macrophages revealed an even distribution throughout the cell cytoplasm (Fig. 1Ei,iii).

### Effect of species-specific LPS on M1 and M2 macrophage cytokine production

Taking into account the differences in TLR4 localization in the context of macrophage polarization, we then challenged M0, M1, and M2 macrophages with LPS derived from three common gram-negative commensals, but also known opportunistic-pathogenic bacteria; *E. coli* (O127:B8), *S. enterica* (Serotype Typhimurium) and *P. aeruginosa* (serotype 10.22). Based on our previous work, outlining the ability for cells to discriminate between LPS serotypes, we hypothesised that commensal bacteria and their associated LPS, could stimulate TLR4 in a varying manner^20^.

**Figure 2:**
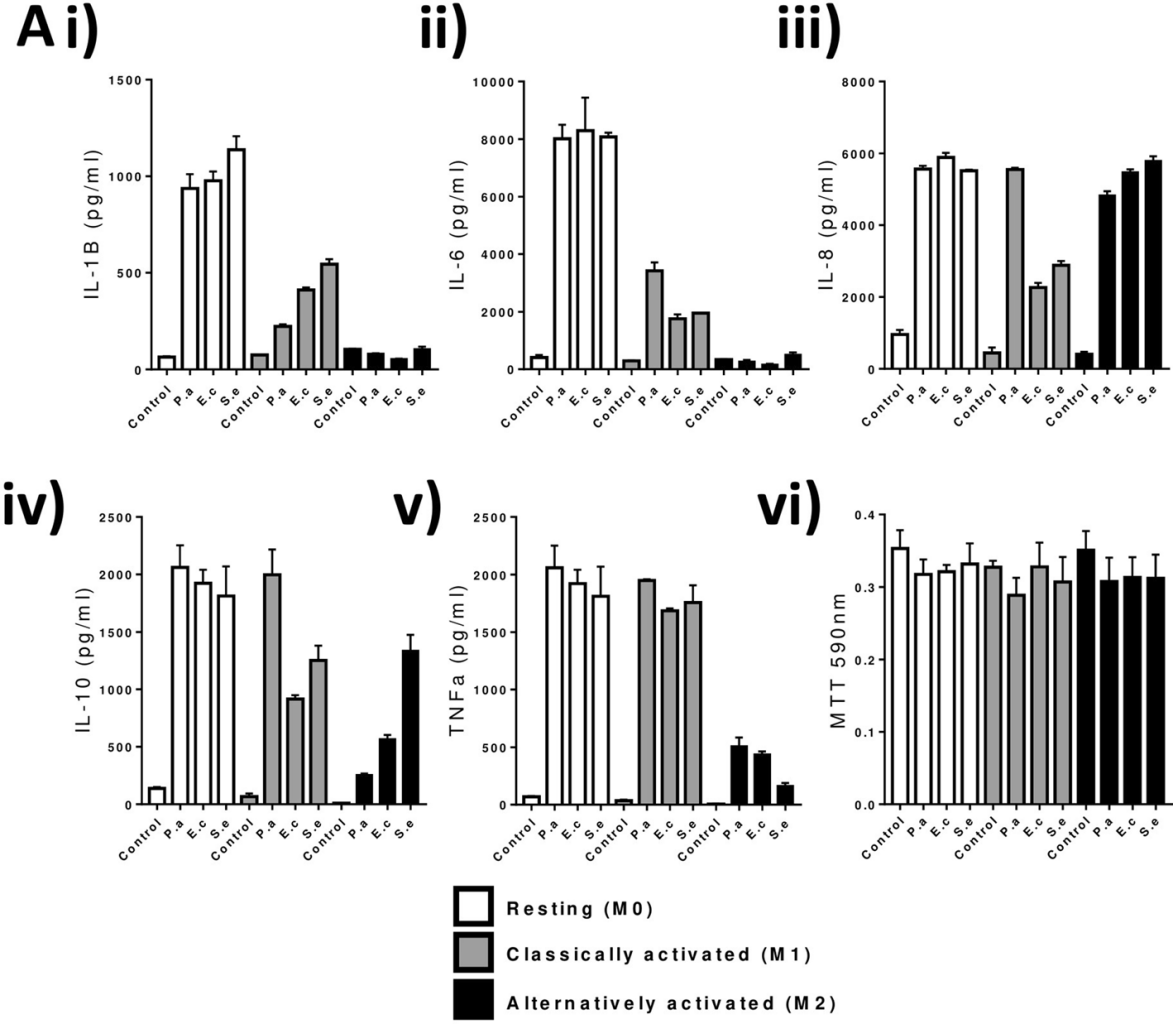
Differential M1 and M2 macrophage de novo NF-κB and IRF3 induction and cytokine production in response to LPS species. PMA differentiated THP-1 monocytes (Resting M0-Macrophages) were stimulated for 24hrs with either (50 ng/ml) recombinant human IFNγ (M1 polarization) or 25 ng/ml each of recombinant human IL-4 and IL-13 (M2). Macrophages were challenged with 1 ng/ml LPS in complete media for up to 24 h after which supernatants were collected for (**Ai**–**v**) multiplex cytokine (Il-1B, IL-6, IL-9, IL-10, TNFα), (**vi**) representative MTT viability assay experiment. Data is expressed as the mean ± SEM of 3 independent differentiations of THP-1 derived macrophages of passages 5–8. Mean ± SD is displayed for MTT. Statistical analysis performed on qPCR data was a multiple unpaired students t-tests comparing stimulated to control, **P < 0.01, ***P < 0.001, ****P < 0.0001.

We report here that induction of inflammatory mediators: IL-1β, IL-6, IL-8, IL-10 and TNFα was significantly altered through the stimulation of these macrophages with LPS from *P. aeruginosa, E. coli* or *S. enterica* (Fig. 2A).

### *S. enterica* LPS stimulates TLR4-IRF3 within the endosome

**Figure 3:**
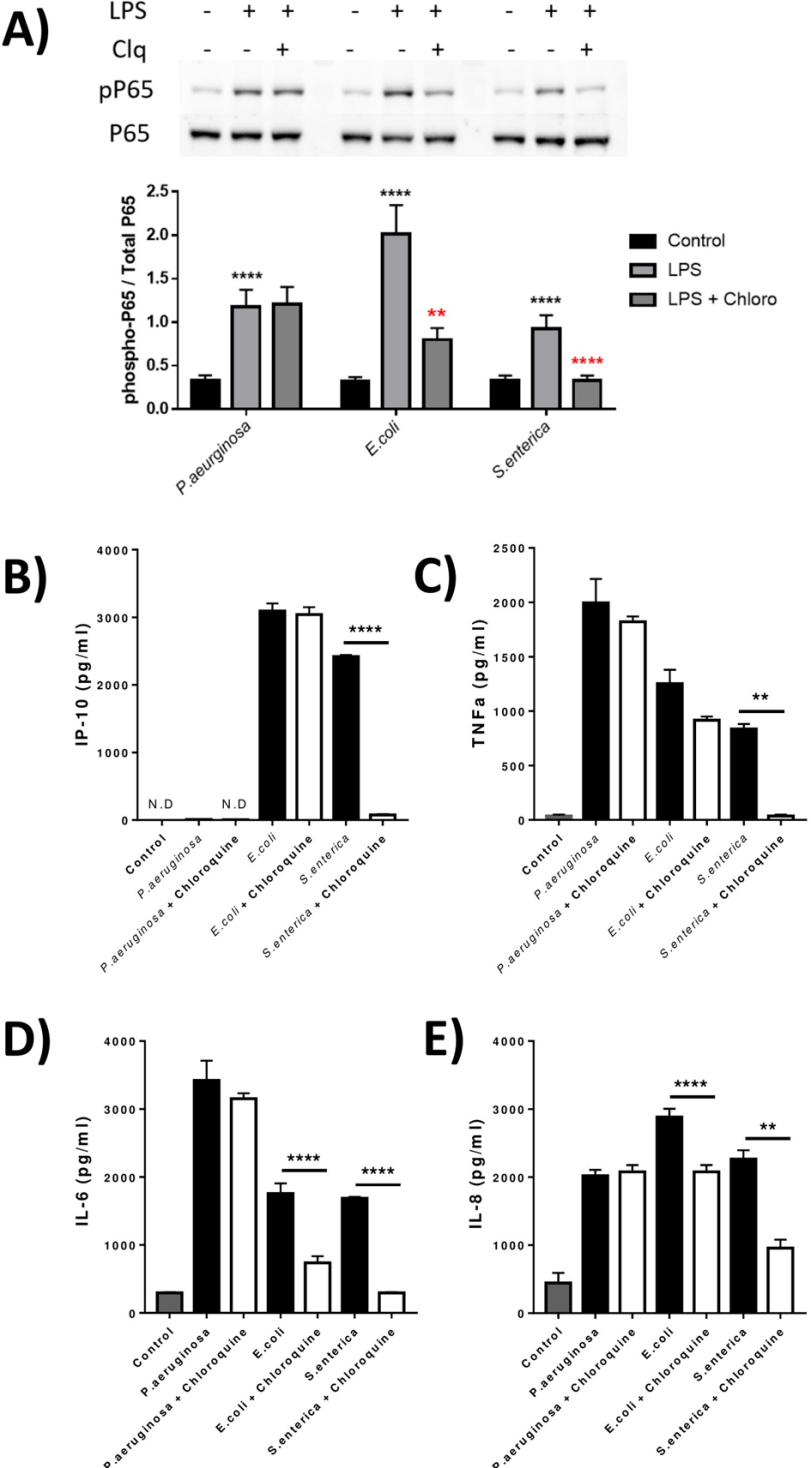
*E. coli* and *S. enterica*, but not *P. aeruginosa* LPS signal partially via the endosome in polarized macrophages resulting in altered inflammatory cytokine production. Differentiated M1 THP-1 macrophages were stimulated with LPS from *P. aeruginosa*, *E. coli* or *S. enterica* with or without pre-incubation with 100 µM Hydroxy-Chloroquine. Induction of (**A**) LPS induced P65 phosphorylation measured after 30 min with/without Clq (Hydroxychloroquine). (**B**–**E**) IP-10, TNFα, IL-6 and IL-8 production was measured in conditioned supernatants. Data is expressed as the mean ± SEM of 3 independent differentiations of THP-1 derived macrophages of passages 5–8. Statistical analysis performed on qPCR data was a one-way ANOVA with Tukeys posthoc test, **P < 0.01, ****P < 0.0001.

Through the presence of TLR4 in intracellular regions of M1 macrophages (Fig. 1), we hypothesised that the response to LPS of intracellular pathogens such as *S. enterica* and/or *E. coli* could be directed through an endosomal-dependent pathway. In order to investigate whether TLR4 was actively endosomal-bound and activated by endosome acidification, we prevented the latter through treatment with hydroxychloroquine. Downstream phosphorylation of P65 demonstrated that both *E. coli* and *S. enterica* LPS, but not that of *P. aeruginosa,* were signalling through the endosome compartment (Fig. 3A). Only *E. coli* and *S. enterica* LPS induced: IP-10 synthesis which was significantly ablated when treated with hydroxychloroquine (Fig. 3B). All LPS tested induced TNFα, IL-6 and IL-8 production, as measured by ELISA. This production was significantly reduced in *E. coli* and *S. enterica* LPS stimulated cells with the addition of hydroxychloroquine, indicating the production of TNFα, IL-6, and IL-8 was driven by IRF3 (Fig. 3C–E). LPS derived from *P. aeruginosa* activated only P65 phosphorylation in a canonical fashion and thus, was unaffected by treatment with hydroxychloroquine in all parameters tested. These data overall show that *E. coli* and *S. enterica* LPS can activate endosomal-bound, or endosomal-trafficked TLR4, inducing an IRF3-driven inflammatory response, whilst *P. aeruginosa* LPS signals via cell surface TLR4-driven response, independent of the endosome.

### Biased signalling of LPS in M1 macrophages is TAK1 and TBK1 dependent

Although we cannot answer here, whether a purified product such as LPS has additional processing by the endosome allowing it to be functionally active, we can state that its activation is important in the TLR4-LPS recognition. The pathway whereby TLR4 was being activated by different species of LPS was the next question. Therefore, we focused our investigation on M1 macrophages due to the compartmentalization phenomenon of TLR4 and the ability to discriminate between LPS signalling non-canonically through the endosome (Fig. 2). In order to further elucidate whether TLR4 signalling was individually-biased to bacterial LPS in this scenario, we utilised pharmacological interventions to alter TLR-specific intracellular signalling proteins mitogen-activated protein kinase kinase kinase 7 (MAPK7 also known as TAK1) and TANK-binding kinase 1 (TBK1). TAKANIB and MRT67307 have been well documented to prevent activation of these kinases in a highly-selective and controlled manner, thus we sought to use these agents to distinguish the activation pathways of TLR4, and ultimate phosphorylation of p65 or IRF3, respectively^21,22^.

**Figure 4:**
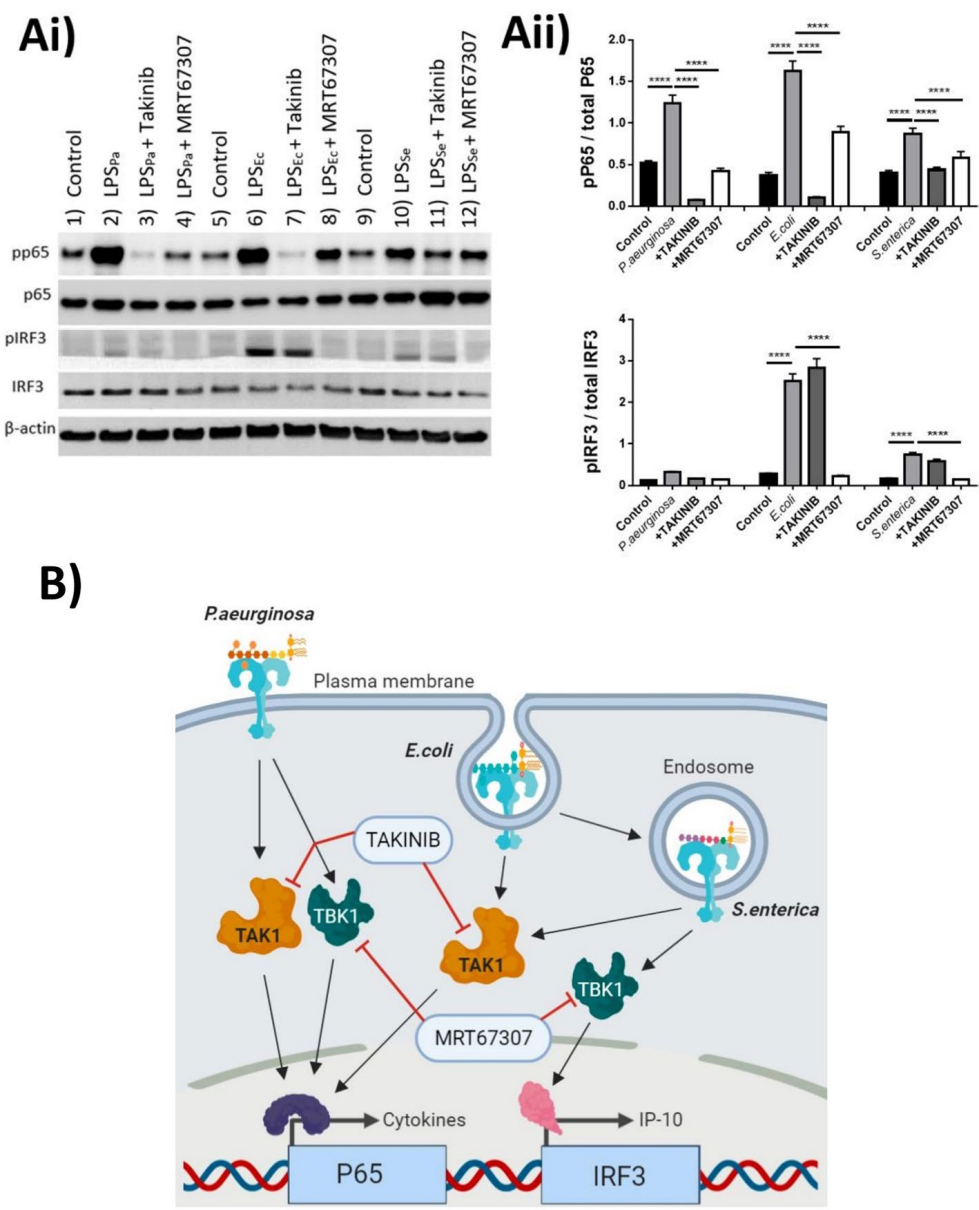
LPS-driven phosphorylation of P65 and IRF3 is species specific and TAK1 and/or TBK1 dependent. Cells were then lysed and assayed for (**Ai**) phospho-P65, total p65, phospho-IRF3, total IRF3 and B-Actin acting as a loading control (Upper panel is a representative of n = 3 experiments). (**Aii**) Phosphorylated P65 and IRF3 were analysed in comparison to total P65 or IRF3 respectively. Data is represented as mean ± SEM of 3 separate stimulations performed on IFNγ differentiated M1 macrophages Passage 6–9. (**B**) Graphical representation of proposed signalling dynamics of the individual LPS via TLR4 and targets for TAKINIB or MRT67307 inhibitors. Cells were stimulated with LPS (1 ng/ml) with or without 1 h pre-treatment with TAKINIB (10 µM) or MRT67307 (10 µM) for 30 min in serum starved conditions. Membranes were stripped and re-probed, or cut longitudinally, to probe multiple antibodies within the same membrane, full-uncropped images can be found in Supplemental Fig. S1. The images in figure are from 2 separate gels and membranes. Statistical analysis performed on qPCR data was a one-way ANOVA with Tukeys posthoc test **P < 0.01, ***P < 0.001, ****P < 0.0001. Illustration created using Biorender.com. Figure generated by MS with permissions granted for publication.

Treatment of classically activated M1 macrophages with LPS induced phosphorylation of p65 and IRF3 (Fig. 4A). Interestingly, and in concordance with our previous data, p65 phosphorylation was induced by all LPS tested (Fig. 4Ai), while IRF3 phosphorylation was driven only by *E. coli* and *S. enterica* (Fig. 4Aii). Inhibition of TAK1 and TBK1 respectively revealed a clearer biased pathway. *P. aeruginosa* LPS signalled through both TAK1 and TBK1 induced phosphorylation of p65, *E. coli* and *S. enterica* however, only signalled through TAK1 resulting in P65 phosphorylation, and through TBK1 exclusively for IRF3 phosphorylation. With the previous data from Fig. 3, we can also suggest that the TBK1-IRF3 axis is likely mediated through the endosomal activation of TLR4 in both *E. coli* and *S. enterica* LPS stimulated cells (Graphical depiction: Fig. 4B).

## Discussion

Unlike the other members of the TLR superfamily, TLR4 is the only currently known receptor member to possess the ability to trigger both MyD88-dependent and -independent pathways after ligand binding^23^. MyD88-dependent signalling culminates primarily in the activation of NF-κB acting as a potent inducer of inflammation, whilst MyD88-independent pathways mainly activate IRF3 regulatory genes promoting anti-viral type-1 interferons. This ability of TLR4 to signal via each of these mechanisms was seen by many as an artefact, having had little evidence suggesting a ligand definitive reason to why this would occur but being aware of its necessity^24^. Our results first demonstrate that biased signalling between MyD88-dependent and -independent pathways accounts for differences in responses to species-specific LPS, specifically LPS from intracellular pathogens such as *S. enterica* and to a lesser extent forms of *E. coli*. Second, using differentiated THP-1 human macrophages, our findings suggest that polarization leads to differential expression and localisation of TLR4 on and within the cell, thereby allowing a physical discrimination for signalling. In particular, we demonstrate that M1 macrophages (classically activated inflammatory macrophages) expressed TLR4 within intracellular compartments after differentiation and these receptors were confirmed to be activated by an endosomal mechanism for the recognition of *S. enterica* LPS leading to a MyD88-independent IRF3 cascade. Third, we establish that differential activation of TLR4 within both M1 and M2 macrophages by species-specific LPS, have distinct consequences on gene transcription as well as cytokine and chemokine production. At this time, we recognize that the use of an artificial polarization system such as PMA-differentiation within these cells may not represent the true extent of signalling pathway. The IL-1β, for instance, usually requires a two-step, caspase-1 dependent signalling system for cleavage and secretion. However, it is documented that non-canonical inflammasome (caspase 4/5) can cleave IL-1β if LPS has contaminated the cytoplasmic compartment, perhaps suggesting a higher phagocytic capacity of the differentiated M0 and M1 macrophages^25^.

Due to the broad diversity of TLR4-ligands, including microbe-associated molecular patterns (MAMPs), such as LPS, the range of variation between the recognition of gram-negative bacteria within the host can be drastically different. The consequence of these structural variations during the progression of infection and disease is currently being widely investigated by many groups with interesting results however, a definitive consensus is still lacking^26,27^. Investigation of the role these varied structures play in infection and autoimmune inflammation is an area of great scientific interest, as are TLRs as therapeutic targets for interventions during infection and disease^28^. The evidence is clear that TLRs can respond to ligands in a dose-dependent manner, but having an ability to differentiate between different structural moieties found on individual pathogens is truly a magnificent piece of evolutionary design.

Overall, exogenous molecules such as LPS enter cells through recognizing cell surface receptors, such as TLR4, activating downstream intracellular responses. Endocytosis of cell surface receptors is a characteristic of macrophages used for the phagocytosis of bacteria or particulates for lysosomal destruction; or in some cases internalised for presentation to intra-cellular receptors^29^. Utilising this pathway, innate immune cells, such as macrophages, can internalise microbial products and allow presentation to intracellular receptors for alternate pathway activation. TLR4 internalisation and signalling through a MyD88-independent mechanism, rather than the classical MyD88-dependent pathway, has been previously described^4^. However, our findings suggest that classically activated, M1 macrophages, internalised expression TLR4 is discriminant toward distinct forms of LPS, as exemplified by how *E. coli*-, *S. enterica*-, and *P. aeruginosa*-derived LPS is recognised and acted upon.

We have assessed and outlined a hypothesis by which pro-inflammatory macrophages, i.e. those exposed to a highly PAMP/DAMP rich environment, internalise their TLR4s as perhaps a mechanism to reduce their sensitivity to the surrounding milieu. However, they maintain intracellular expression in order to promote a response if they become infected by pathogenic intracellular bacteria such as *S. enterica.* Alternatively-activated M2 macrophages however, favour the expression of TLR4 on the cell surface during the reparative stage, perhaps in an effort to sense the remaining microbial- and damage-products to promote the necessary cytokines for wound healing. LPS from diverse bacteria have been shown to activate distinct MyD88-dependent and -independent pathways in human glioma cells and murine macrophage, although the discriminative capacity was not fully understood^24,30^. Similar to our studies, both papers demonstrated the MyD88-independent activation of TLR4 induced by *S. enterica.* Interestingly, this discriminative capacity could be adapted and developed to deplete and/or modulate TLR4-signalling pathways in a controlled manner, thereby eliminating NF-κB and/or IRF signalling in order to better study the overarching effects of the stimulation for therapeutic research^31^.

A shift in balance of LPS-TLR4 signalling from unbiased NF-κB/IRF3 toward the more anti-inflammatory IRF3 cascade is a known feature in regulating tolerance to LPS^32^. The role of this balance shift in chronic diseases such as: inflammatory bowel disease, amyotrophic Lateral Sclerosis, liver disease, or rheumatic diseases, is still poorly understood, but creates an avenue of research for future investigation especially in diseases where there is known involvement of bacterial products and/or NF-κB/IRF3 signalling^15,33–35^. In the expanding realm of the microbiota and its involvement in disease progression, we theorise that this discriminative capacity may be defunct in certain patient cohorts and infections. In conclusion, our data demonstrate the capability of cytokine-differentiated macrophages to distinguish and respond to LPS from specific species in a ligand-dependent manner. We demonstrate the biased and unbiased signalling of LPS by these cells and how polarity of macrophages alters their ability to detect *S. enterica* through the redistribution of TLR4. Notably, these differences can be further seen whereby the downstream signalling can be separated into TAK1-dependent and TBK1-dependent mechanisms. With these findings we hope to be able to investigate more clearly the role of bacterial-host interactions in the aspect of endotoxin signalling.

## Conclusions

Innate immune cells such as macrophages, play a critical role in the first response to infection through expression of a wide array of pattern recognition receptors, robust cytokine and chemokine production, and a phagocytic capacity. We have discovered that with compartmentalization of TLR4, polarized macrophages can discriminate between LPS of different origins and invoke specific responses as a consequence. These findings help to resolve an understanding of biased TLR4 signalling in response to LPS and opens up new avenues for TLR4 as a therapeutic intervention strategy during bacterial infection and also chronic inflammation during microbial dysbiosis.

## Supplementary Information

Supplementary Information.

## Abbreviations

TLR: Toll-like receptor
MyD88: Myeloid differentiation primary response 88
TRIF: TIR-domain-containing adapter-inducing interferon-β
NF-κB: Nuclear factor kappa-light-chain-enhancer of activated B cells
IRF3: Interferon regulatory factor 3
LPS: Lipopolysaccharide
M1: Classically activated macrophage
M2: Alternatively activated macrophage
TAK1: Mitogen-activated protein kinase kinase kinase 7
TBK1: TANK binding kinase 1
PMA: Phorbol 12-myristate 13-acetate
IFNγ: Interferon gamma
IL: Interleukin
IP-10/CXCL10: Interferon gamma-induced protein 10
CCR7: Chemokine receptor 7
CD206: Mannose receptor C type 1
CD163: Haemoglobin scavenger receptor
CCL17: Chemokine (C–C motif) ligand 17
