# Supplementary material for: Ultra-purification of Lipopolysaccharides reveals species-specific signalling bias of TLR4: importance in macrophage function
Source: Sci Rep. 2021 Jan 14;11:1335. doi: 10.1038/s41598-020-79145-w (PMC7809447; doi:10.1038/s41598-020-79145-w)
Supplement: Supplementary file 1 — Supplementary Information. [file 41598_2020_79145_MOESM1_ESM.docx]

**Ultra-purification of Lipopolysaccharides reveals species-specific signalling bias of TLR4 – importance in macrophage function.**

^1^Matthew Stephens, ^2^Shan Liao & ^1^Pierre-Yves von der Weid

Inflammation Research Network, Snyder Institute for Chronic Diseases,

^1^Department of Physiology and Pharmacology, Cumming School of Medicine, University of Calgary, Alberta, Canada.

^2^Department of Microbiology, Immunology & Infectious Disease, Cumming School of Medicine, University of Calgary, Alberta, Canada.

Correspondance:

Pierre-Yves von der Weid

[vonderwe@ucalgary.ca](mailto:vonderwe@ucalgary.ca)

**Supplemental figure 1 (full western blot images for Figure 3):**

For publication, images were cropped to show only the bands of interest within Figure 3, contrast and brightness were not altered in the image. The membranes were stained for phosphorylated p65, stripped and re-probed for total p65 only once to prevent excessive protein loss. All blots were exposed for detection for 60s and sizes of bands validated against the predicted band size and PageRuler Prestained Protein Ladder (Biorad, USA).


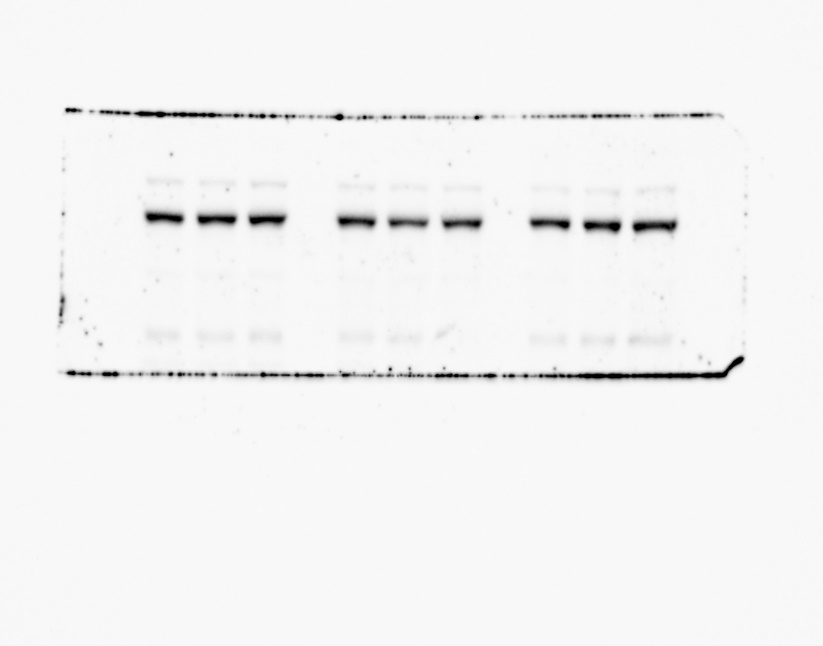
**Total P65**

**Phosphorylated P65**


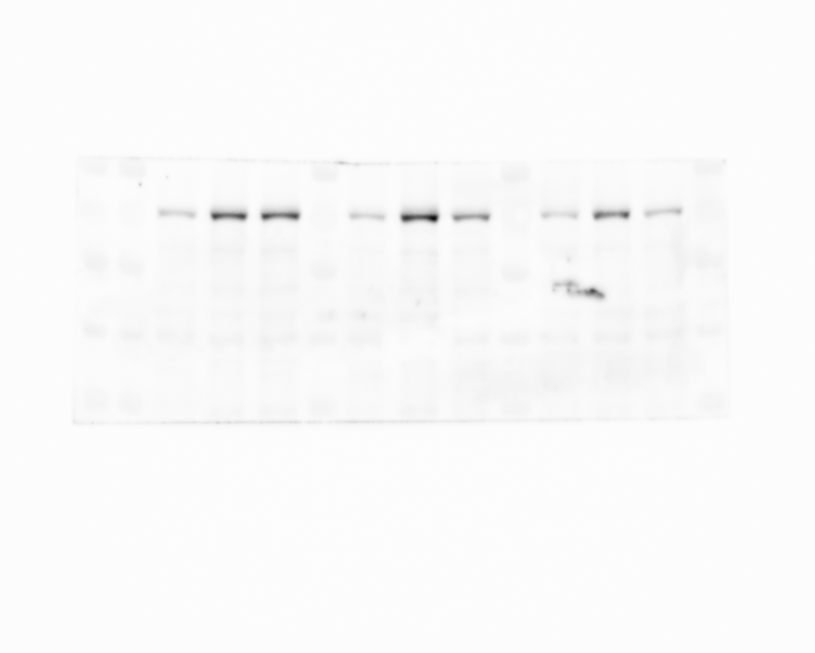


**Supplemental figure 2 (full western blot images for Figure 4):**

For publication, images were cropped to show only the bands of interest within Figure 4, contrast was altered, as was brightness, in a linear fashion by 20% uniformly across the image. Membranes stained for pIRF3 or pP65 were stripped and re-probed a single time to prevent excessive protein loss. Some blots were also cut longitudinally to probe for multiple proteins without interference or spurious results as is commonly accepted. All blots were exposed for detection for 60s and sizes of bands validated against the predicted band size and PageRuler Prestained Protein Ladder (Biorad, USA).

**pIRF3**

**
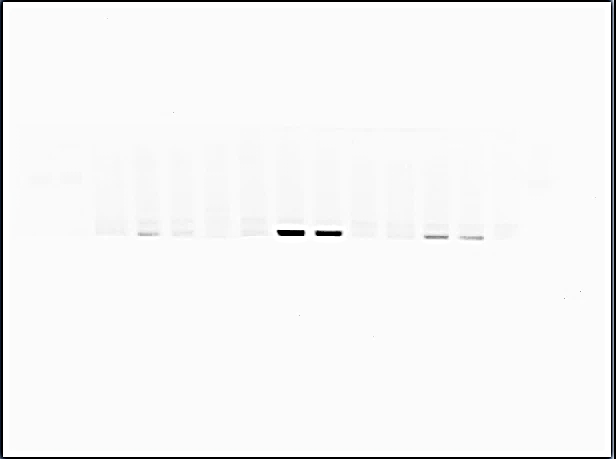
**

**IRF3**

**
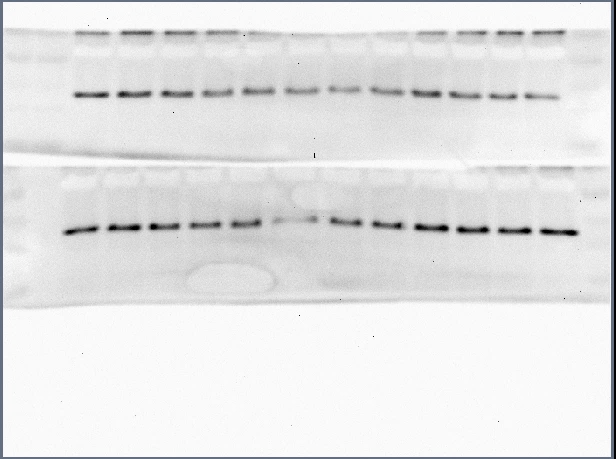
**

**pP65**


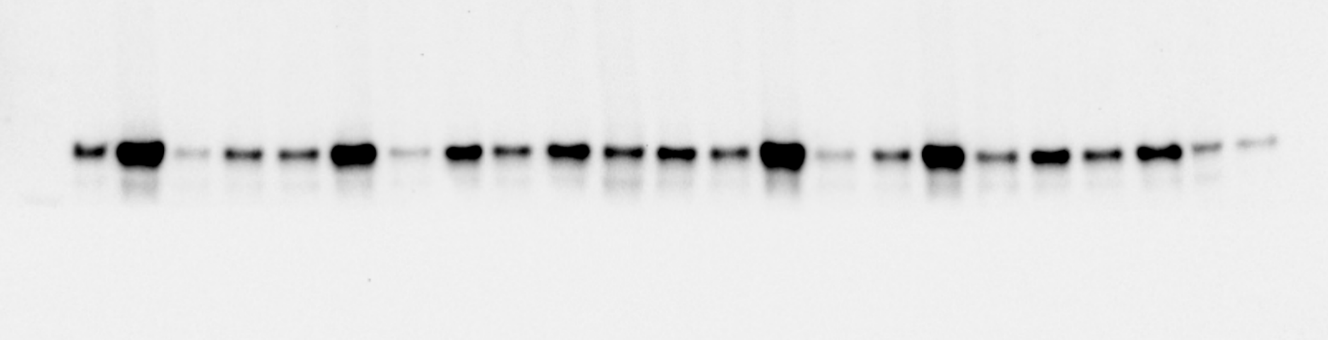


**P65**

**
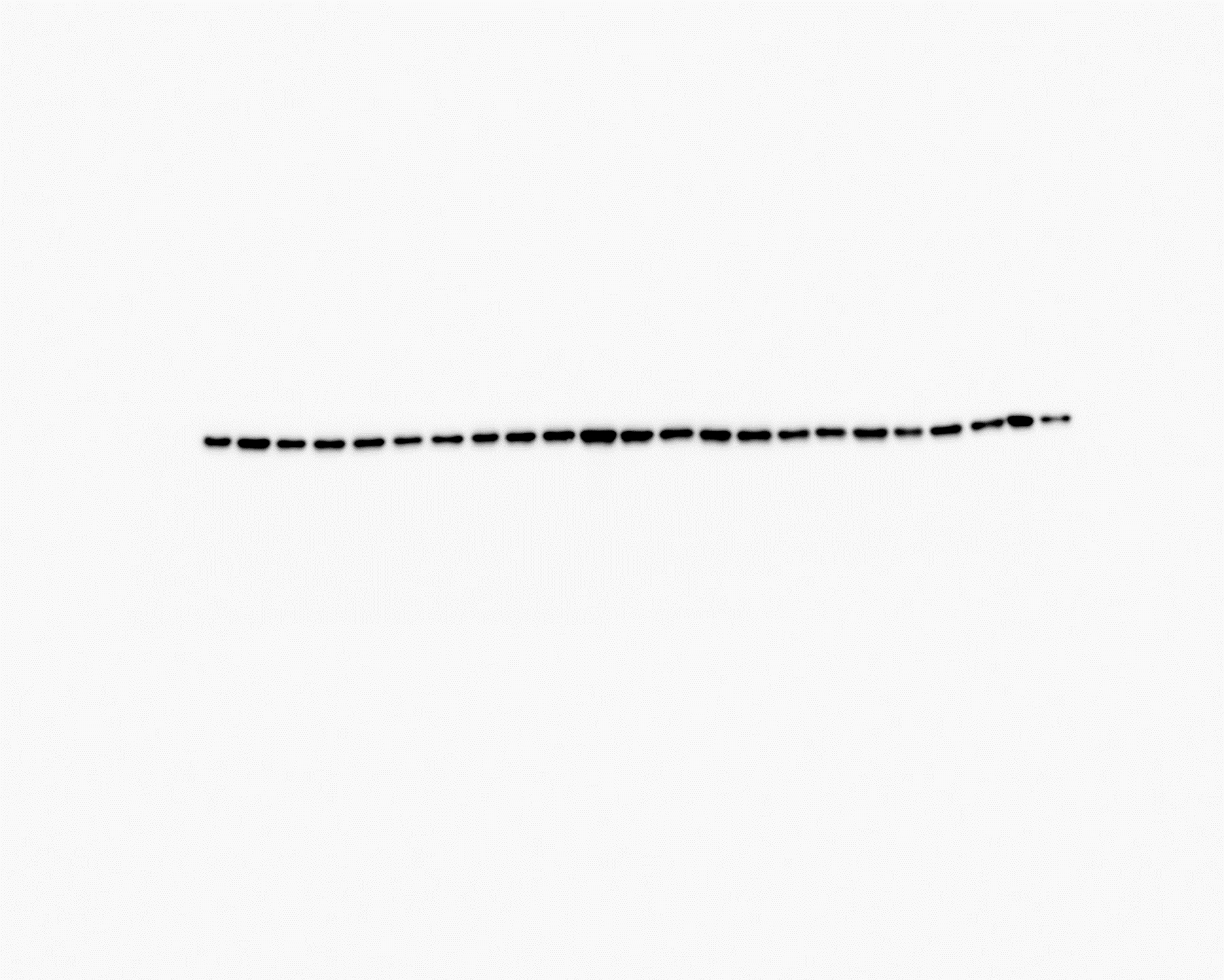
**

**B-actin**

**
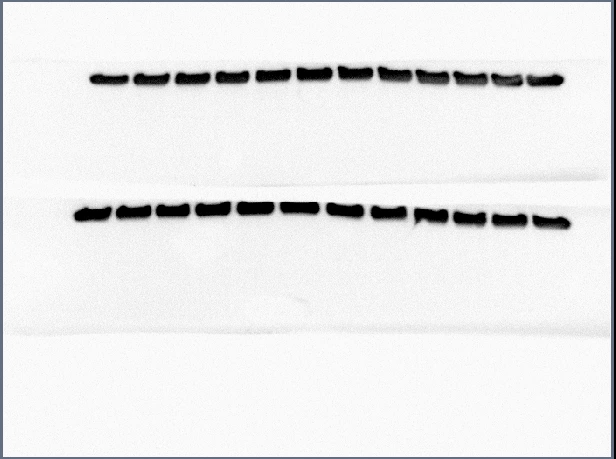
**
